# Evidence of Stress Development as a Source of Driving Force for Grain-Boundary Migration in a Ni Bicrystalline TEM Specimen

**DOI:** 10.3390/ma13020360

**Published:** 2020-01-12

**Authors:** Sung Bo Lee, Jinwook Jung, Heung Nam Han

**Affiliations:** Department of Materials Science and Engineering and Research Institute of Advanced Materials (RIAM), Seoul 08826, Korea; jjw9101@snu.ac.kr (J.J.); hnhan@snu.ac.kr (H.N.H.)

**Keywords:** nickel, transmission electron microscopy (TEM), grain-boundary migration, stress development, stacking fault

## Abstract

In a previous study, using high-resolution transmission electron microscopy (HRTEM), we examined grain-boundary migration behavior in a Ni bicrystal. A specimen for transmission electron microscopy (TEM) was prepared using focused ion beam. The Ni lamella in the specimen was composed of two grains with surface normal directions of [1 0 0] and [1 1 0]. As the lamella was heated to 600 °C in a TEM, it was subjected to compressive stresses. The stress state of the Ni lamella approximated to the isostress condition, which was confirmed by a finite element method. However, the stress development was not experimentally confirmed in the previous study. In the present study, we present an observation of stacking faults with a length of 40–70 nm at the grain boundary as direct evidence of the stress development.

## 1. Introduction

Grain boundaries (GBs) are driven to migrate by various driving force sources, such as the GB energy, the surface energy anisotropy, the stored deformation energy, the chemical driving force, and the elastic energy [1]. Especially for elastically anisotropic materials, inhomogeneities in the elastic strain energy are crucial for GB migration, which was theoretically investigated in Cu GBs by molecular-dynamics (MD) simulations [2] and by phase field simulations [3] and experimentally studied for a Ni GB by Lee et al. [4]. Both Cu and Ni elastically anisotropic, whose Zener’s anisotropy factors $\left[2\left(S_{11}-S_{12}\right)/S_{44}\right]$ at 300 K amount to ~3.2 and ~2.2, respectively, where the elastic compliances, $S_{11}=14.838$, $S_{12}=-6.214$, and $S_{44}=13.193$ (in TPa^−1^) for Cu [5] and $S_{11}=6.524$, $S_{12}=-2.385$, and $S_{44}=7.942$ (in TPa^−1^) for Ni [6].

Using an MD method, Schönfelder et al. [2] analyzed GB migration in a Cu bicrystal with two flat high-angle twist GBs, which thus enabled to exclude the possible source from GB curvature. They found that the elastic driving force was equal to the difference in the elastic energy density across the boundary [2]. Using a phase field model, Tonks et al. [3] also analyzed a Cu bicrystal under an applied strain similar to that studied by Schönfelder et al. [2]. Two cases were considered: One case in which the strain was uniform throughout the bicrystal (isostrain), and the other in which the strains were heterogeneous (heterogeneous strain) with the average strain in the bicrystal equal to the applied strain. 

Under the isostrain condition, the elastically softer grain had a lower strain energy density and it, thus, grew [3]. In contrast, under the heterogeneous-strain condition, the softer grain had higher strain energy, but it grew [3]. In this case, as the softer grain grew, the strain energies of both grains were calculated to be reduced. Therefore, the growth of the softer grain was explained in terms of the total strain energy reduction.

Lee et al. [4] examined the migration of a Ni bicrystalline GB by in situ high-resolution transmission electron microscopy (TEM). A specimen for TEM was prepared using focused ion beam (FIB). A Ni lamella in the specimen was composed of two grains with surface normal directions of [1 0 0] and [1 1 0]. According to a finite element method (FEM), the stress state of the Ni lamella approximated to the isostress condition [4]. The FE analysis showed that under the isostress condition, the total strain energy reduction favored the growth of the [1 0 0] grain. However, the stress development was not experimentally confirmed in the previous study. In the present study, we present an observation of stacking faults (SFs) with a length of 40–70 nm at the GB as direct evidence of the stress development. The long length of SFs observed is discussed in terms of the stress dependence of partial dislocation separation.

## 2. Materials and Methods 

### 2.1. Specimen Fabrication and Observation Method 

**Figure 1:**
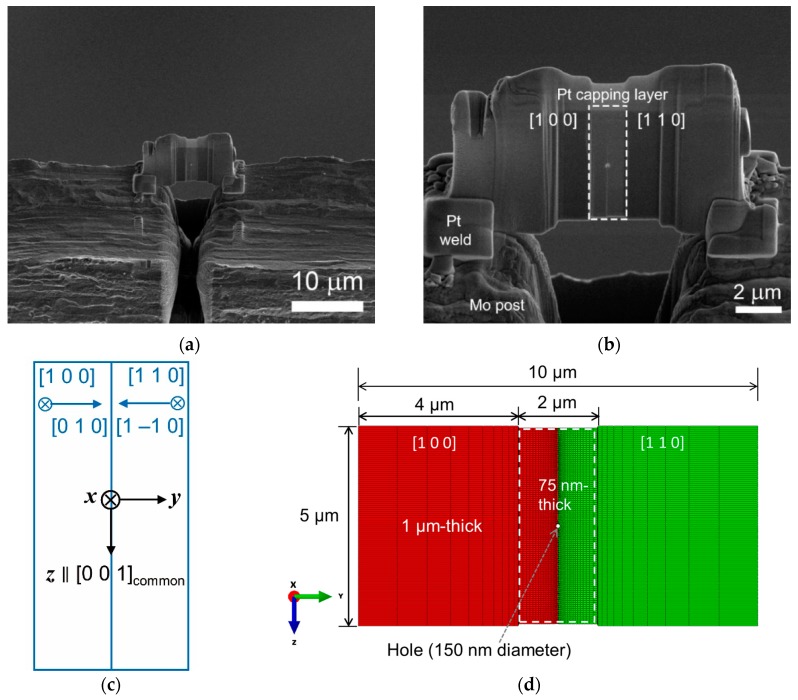
(**a**) Scanning electron microscopy image of a typical focused ion beam (FIB) specimen examined in the present work; (**b**) higher magnification of (**a**); (**c**) crystallographic orientations of the Ni lamella; and (**d**) Finite element model for the heating experiment with similar dimensions of the Ni lamella of the FIB specimen.

The Ni bicrystal used (99.999% purity, MaTeck, Jülich, Germany) had a nominal misorientation relationship of [0 0 1]/45°, where the GB plane was roughly parallel to the [0 1 0] and [1 −1 0] planes of two mating grains. Cross-section specimens for TEM were prepared from the bicrystal on FIB workstations [Nova 200 NanoLab and Quanta 3D FEG (FEI, Eindhoven, Netherlands)]. As shown in Figure 1a, a Ni lamella was welded to an Omniprobe Mo lift-out grid by Pt. The Ni lamella was composed of two grains with different surface normal directions of [1 0 0] and [1 1 0], consisting of thick (1 μm thick) and electron-transparent (75 nm thick, indicated by a dashed box) parts (Figure 1b). The surface normal directions of the two grains are designated as the *x* direction and the GB plane normal direction as the *y* direction (Figure 1c). The [0 0 1] direction, which is common to both grains, is designated as the *z* direction (Figure 1c). The hole shown in Figure 1a,b was drilled to investigate effects of a pinning point on the migration of the Ni bicrystalline GB, which was the aim of [4]. The point is outside the scope of the present study. For further details, refer to [4]. The prepared specimens were heated with the electron beam off to 600 °C at a heating rate of 20 °C/min at a TEM. For further details of the dimensions and orientations in the specimen and TEM observations, refer to [4].

### 2.2. Finite Element Analysis (FEA) of the Ni-Bicrystal Lamella Prepared by FIB

The stress-strain state of the electron-transparent part (indicated by a dashed box in Figure 1b) of the Ni lamella in the FIB specimen was simulated by FEM using ABAQUS/Standard 2018 [4]. Figure 1d shows the FE model used, which had similar dimensions to those of the Ni lamella with the same crystallographic orientations as the Ni lamella (Figure 1b,c). Dimensions of the electron-transparent region were set to 2 μm by 5 μm (width by height) for the FEA.

The FE model (Figure 1d) was composed of 56010 of three-dimensional 8-node coupled temperature-displacement elements (C3D8T). Finer elements were used near the GB to extract more accurate values and to minimize errors. The elastic constants of Ni were adopted from calculation results of Wang et al. [6]. The coefficient of thermal expansion (CTE) was set to 8.6 × 10^−6^ K^−1^. As the FIB specimen was heated to 600 °C, both the Ni lamella and the Mo post would expand. Thus, the actual CTE of the Ni lamella would not simply be the CTE of Ni (13.4 × 10^−6^ K^−1^) but was likely to be close to the difference between the CTE of Ni and that of Mo (4.8 × 10^−6^ K^−1^). This is the reason why the value of 8.6 × 10^−6^ K^−1^ was used for FEA.

The boundary conditions imposed on the model were as follows. The temperature of all nodes was uniformly increased from 25 to 600 °C. Nodes on both ends of the model were constrained from moving along the direction normal to the surface (1 μm by 5 μm (thickness by height)) of both ends to reflect the circumstance that the expansion of the Ni lamella was limited by the Mo post. A boundary condition was also set to avoid rigid body translation and rotation of the model.

**Figure 2:**
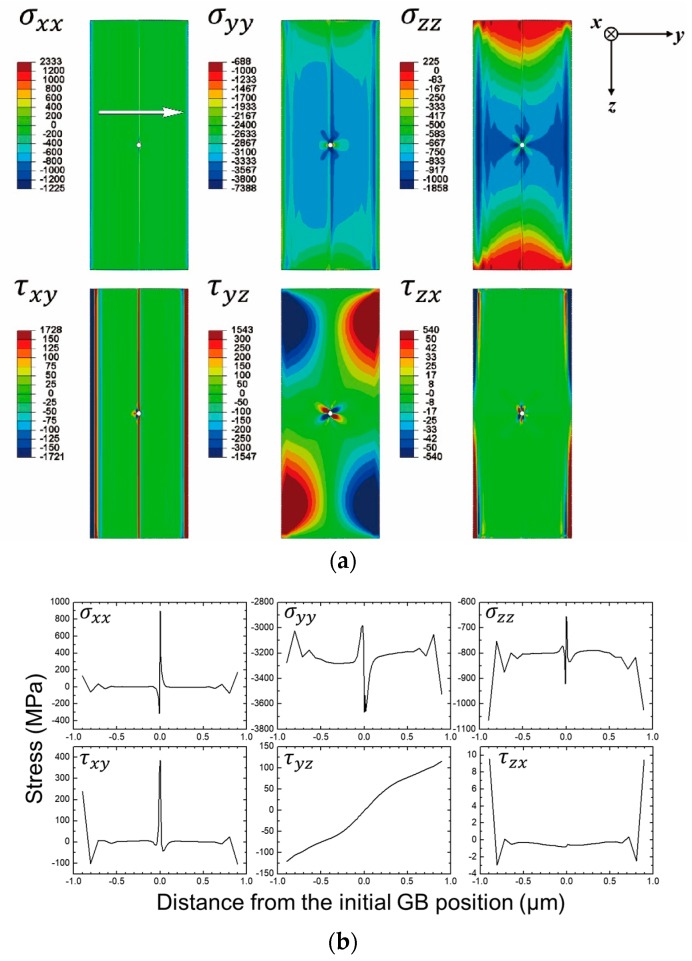
(**a**) Stress maps for the normal and shear components and (**b**) corresponding plots. The arrow in the map of $\sigma_{xx}$ indicates the data export points for the plots, which also applies to the plots in Figure 3. Reprinted from Ref. 4 with permission; Copyright Springer 2019.

**Figure 3:**
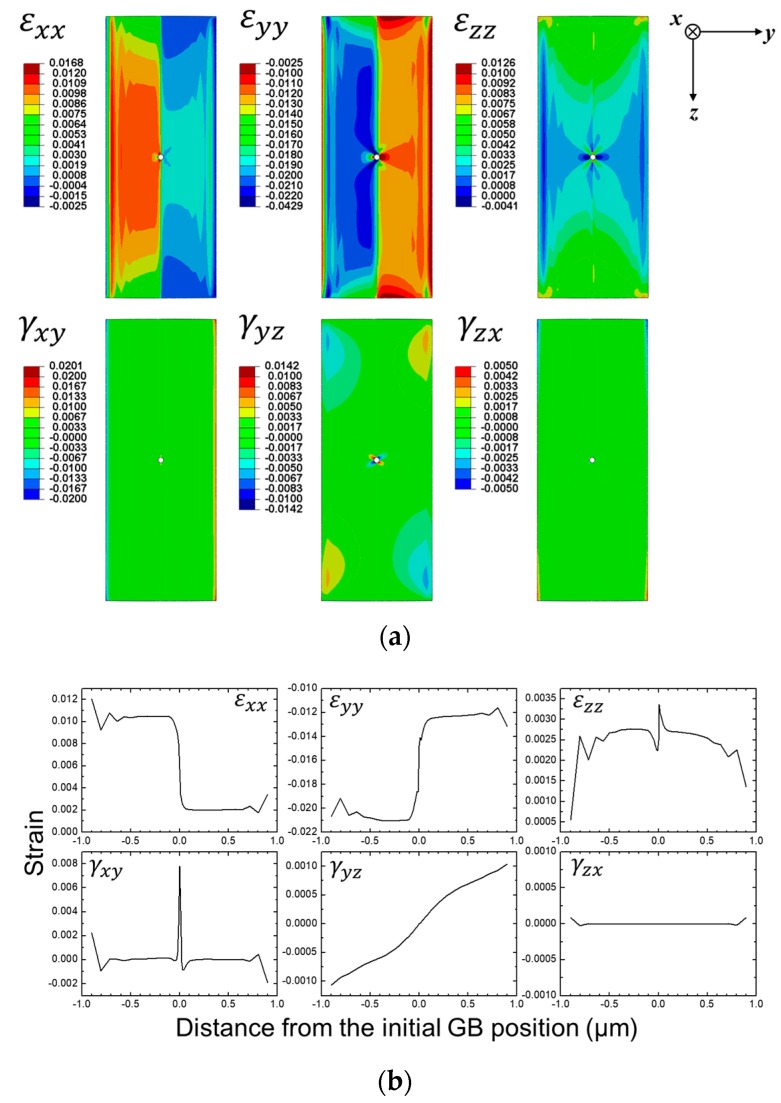
(**a**) Strain maps for the normal and shear components and (**b**) corresponding plots. Reprinted from Ref. 4 with permission; Copyright Springer 2019.

Therefore, the stress state of the Ni lamella approximates to the isostress condition, i.e., the two grains in the lamella uniformly receiving the same stress as the total stress. The stress-strain state of the thin, electron-transparent part of the Ni lamella at 600 °C is expressed by maps and corresponding plots shown in Figure 2 and Figure 3.

Since the thin part of the lamella was very thin as compared with the other dimensions (Figure 1c) and the plane stress state was assumed, $\sigma_{xx}$ acting along the *x* direction (i.e., thickness direction) approximated to 0, which is well reflected in Figure 2. (Henceforth, $\sigma_{ij}$’s mean stress components and $\varepsilon_{ij}$’s mean strain components.) Due to the aforementioned fixed displacement condition, the thin part of the Ni lamella would receive a compressive normal stress in the *y* direction, as well reflected in the FEA (Figure 2). Interestingly, the thin part also had a compressive normal stress in the *z* direction ($\sigma_{zz}$, Figure 2b). This was due to a confinement by the thick part of the lamella. 

Under the isostress condition, the grains received different strains because of their different elastic moduli. For the heterogeneous strain case, the average total strain was constant, but the two grains accommodated the applied strain differently, as simulated by Figure 3. For reference, the present isostress-heterostrain condition corresponds to the second case in Tonks et al.’s study [3].

## 3. Results

**Figure 4:**
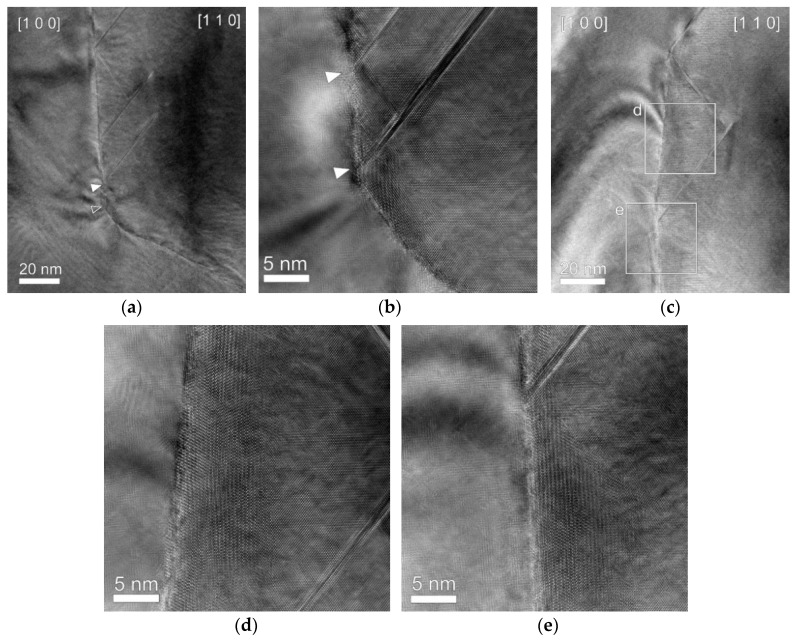
(**a**) Low-magnification image of stacking faults (SFs) pinning the GB and (**b**) enlarged view of (**a**). A SF shown in (**b**) is missing in (**a**), which was taken after (**b**). (**c**) low-magnification image of SFs in another region. (**d**) and (**e**) enlarged views of boxed regions marked as d and e in (**c**).

As shown in Figure 4, SFs with a length of 40–70 nm are observed near the GB. Such long SFs do not form inside the grains. Interestingly, one of two SFs shown in Figure 4b is not seen in a lower-magnification image, as indicated by an open triangle marking the original position of the missing SF in Figure 4a. Figure 4a was taken after Figure 4b. It is considered that the SF disappeared during the elapsed time between the two images. Figure 4c–e was taken from another region of the GB. Close examination of the region between the SFs (Figure 4d,e) reveals moiré patterns bulging from the [1 0 0] grain into the [1 1 0] grain, also demonstrating that the GB is prevented from migrating to the [1 1 0] grain at the tips of the SFs.

## 4. Discussion

The SFs shown in Figure 4 originate from plastic deformation with heating to 600 °C. A SF forms between two partial dislocations, leading and trailing. An equilibrium separation between the two partials is determined by a balance between the repulsive forces of the two partials and the attractive forces due to the relevant SF energy. In Ni, the SF energy is estimated to decrease from 110 mJ m^−2^ at room temperature to 64.3 mJ m^−2^ at 600 °C (as read from a calculated curve from a result by Zhang et al. [7]). At the SF energy of 64.3 mJ m^−2^ [7], the separation distance approximates to ~1 nm without any external stress. The larger separation distances (40–70 nm) shown in Figure 4 are attributed to the applied stress developed during heating in the present work.

**Figure 5:**
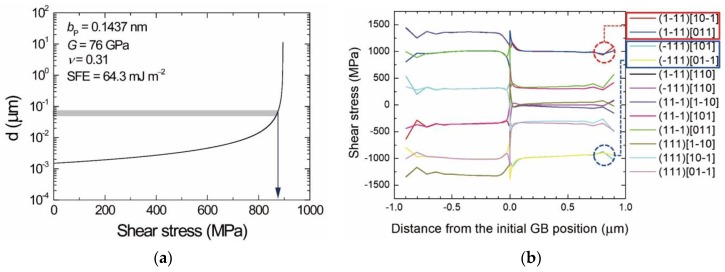
(**a**) Stress dependence of the separation distance for Ni. (**b**) FE calculation of shear stresses acting on slip systems. The inset shows materials parameters of Ni used for the calculation of the stress dependence $(b_{P}$ = magnitude of the Burgers vector of partial dislocations (1/6 〈1 1 2〉), G = shear modulus, ν = Poisson’s ratio, and SFE = stacking fault energy). The components of the shear stresses appear in the legend of (**b**).

Byun [8] related an applied stress to the increase in separation distance between the two partials. He formulated force balance equations for leading and trailing partial dislocations by considering not only the repulsive force between the two partials and the attractive force due to the SF energy, but also the Peach–Koehler force from an applied stress field and the friction force to the glide of the partial dislocations [8]. The calculation results demonstrate that the separation distance increases with applied stress [8]. We have extended Byun’s calculation [8] to our case with the help of FEM and obtained a plot as shown in Figure 5a. Figure 5a indicates that the separation distance of 40–70 nm corresponds to an applied shear stress of ~900 MPa. Shear stresses plotted in Figure 5b are obtained by processing FEA export data in consideration of a total of 12 slip systems in fcc Ni. Boxed slip systems in the legend of Figure 5b, which correspond to the observed SFs, are shown to have enough shear stresses (~1 GPa) for the observed separation distance (Figure 5b). Conversely, the long SFs (Figure 4) are evidence that the stresses acting on the thin part of the Ni lamella have been high enough to produce the strain energy which drives the GB migration.

The nucleation of a full dislocation usually requires a much higher energy than dislocation propagation and thus its heterogeneous nucleation at the GB is the most plausible mechanism of dislocation formation. Normally in a homogenous matrix, the shear stress for the nucleation of a full dislocation is expected to amount to ~*G*/10, where *G* is the shear modulus of the system (76.7 GPa for Ni, see the caption of Figure 5a). Our calculation of Figure 5a reveals the shear stress of maximally 1 GPa acting on the slip systems, which is lower than the expected shear stress required for dislocation nucleation in the homogenous Ni matrix. However, our system is a GB. The GB is imperfect as compared with the homogeneous matrix and would require a much less shear stress for dislocation nucleation. We surmise that full dislocations were nucleated at the GB at much lower shear stresses or were already nucleated during the bicrystal fabrication. It is noted that Figure 5 is regarding the shear stress required for the separation of a full dislocation.

The disappearance of the SFs during imaging at room temperature (Figure 4a,b) is explained as follows: The SFs formed at 600 °C are expected to cause a decrease in volume to reduce the applied compressive stresses ($\sigma_{yy}$ and $\sigma_{zz}$, Figure 2). Therefore, after cooling to room temperature, conversely, both ends of the Ni lamella will apply tensile stresses to the Ni lamella, which can drive SFs to shrink or disappear to further reduce the total strain energy, as shown in Figure 4a. Because no applied stress exists after cooling to room temperature, the long SFs shown in Figure 4 could exist at room temperature probably due to lattice friction.

As shown in Figure 4a,c, the long SFs are always located in the [1 1 0] grain side and their one end contacts the GB. As shown in Figure 5b, some of the slip systems in the [1 0 0] grain also receive shear stresses enough for large separations. Therefore, the consideration of only the shear stress effect is not enough to explain why the long SFs are not observed in the [1 0 0] grain. The observation of one end of the long SFs touching the GB (Figure 4) seems to indicate that the GB can stabilize the SFs. One of two partial dislocations bordering a SF, when contacting a GB, will be more stable than the other located in the matrix lattice because the GB has an open structure. Thus, the case in which one partial touches a GB with the other in the grain interior is likely to be more stable against the disappearance of SFs than the case in which both partial dislocations are located in the grain interior. This is the reason why such long SFs do not form inside the grains. Partial dislocations bordering SFs in the [1 1 0] grain are more probable to meet the GB because the GB was observed to migrate into the [1 1 0] grain [4]. Therefore, SFs in the [1 1 0] grain side will remain long as it was at 600 °C, as shown in Figure 4. While the GB migrated into the [1 1 0] grain, SFs in the side of the [1 0 0] grain, though one end initially touching the GB, would be detached from the GB, which is thus expected to make them unstable. 

## 5. Conclusions

The long SFs observed in the present study have been presented as evidence for the stress development required for the driving force for migration. The present study certainly demonstrates that such a design of bicrystalline GB specimens prepared by FIB for heating experiments at TEMs is very useful for elucidating effects of the applied stress on the GB migration.
